# Estimation of the optimal dosing regimen of escitalopram in dogs: A dose occupancy study with [^11^C]DASB

**DOI:** 10.1371/journal.pone.0179927

**Published:** 2017-06-23

**Authors:** Olivia Taylor, Nick Van Laeken, Ingeborgh Polis, Robrecht Dockx, Lise Vlerick, Andre Dobbeleir, Ingeborg Goethals, Jimmy Saunders, Nele Sadones, Chris Baeken, Filip De Vos, Kathelijne Peremans

**Affiliations:** 1 Department of Veterinary Medicine, Ghent University, Ghent, Belgium; 2 Laboratory of Radiopharmacy, Ghent University, Ghent, Belgium; 3 Department of Psychiatry and Medical Psychology, Ghent University, Ghent, Belgium; 4 Department of Nuclear Medicine, Ghent University Hospital, Ghent, Belgium; 5 Laboratory of Toxicology, Ghent University, Ghent, Belgium; Wayne State University, UNITED STATES

## Abstract

Although the favourable characteristics of escitalopram as being the most selective serotonin reuptake inhibitor and having an increased therapeutic efficacy via binding on an additional allosteric binding site of the serotonin transporter, its dosing regimen has not yet been optimized for its use in dogs. This study aimed to estimate the optimal dosing frequency and the required dose for achieving 80% occupancy of the serotonin transporters in the basal ganglia. The dosing frequency was investigated by determining the elimination half-life after a four day oral pre-treatment period with 0.83 mg/kg escitalopram (3 administrations/day) and a subsequent i.v. injection 0.83 mg/kg. Blood samples were taken up to 12 hours after i.v. injection and the concentration of escitalopram in plasma was analysed via LC-MSMS. The dose-occupancy relationship was then determined by performing two PET scans in five adult beagles: a baseline PET scan and a second scan after steady state conditions were achieved following oral treatment with a specific dose of escitalopram ranging from 0.5 to 2.5 mg/kg/day. As the elimination half-life was determined to be 6.7 hours a dosing frequency of three administrations a day was proposed for the second part of the study. Further it was opted for a treatment period of four days, which well exceeded the minimum period to achieve steady state conditions. The optimal dosing regimen to achieve 80% occupancy in the basal ganglia and elicit a therapeutic effect, was calculated to be 1.85 mg/kg/day, divided over three administrations. Under several circumstances, such as insufficient response to other SSRIs, concurrent drug intake or in research studies focused on SERT, the use of escitalopram can be preferred over the use of the already for veterinary use registered fluoxetine, however, in case of long-term treatment with escitalopram, regularly cardiac screening is recommended.

## Introduction

For many years, selective serotonin reuptake inhibitors (SSRIs) have gained a prominent position in the treatment of mood- and anxiety disorders. Among them, escitalopram, the pharmacologically active S-enantiomer of racemic citalopram, is not only the most selective SSRI available in clinical practice, it can also be classified as an allosteric SSRI. The additional interaction of escitalopram with an allosteric binding site on the serotonin transporter (SERT) modulates the affinity of escitalopram at the primary (orthosteric) site. This results in an increased therapeutic efficacy of escitalopram, which exceeds the effects of an equipotent dose of racemic citalopram [1,2]. Recently, the patent expiration of the brand-name drug in 2012 [3] and the release of generics on the market have lowered the price and opened more perspectives for the use of escitalopram in veterinary medicine. Initial attempts to treat dogs suffering from clinical anxiety disorders resulted in frequent therapeutic failure because they were set up by simply transposing knowledge from man to dog and using the flawed approach to base dose on body weight. As stated by Toutan and colleagues, the main challenge in veterinary medicine is however not to select a drug, but rather to determine a rational dosing regimen as this is based on multiple factors such as the species anatomy, its biochemistry, physiology, etc. [4]. As such, high interspecies differences are reported for substances having a molecular weight (MW) between 300 and 600–800 g/mol, which encloses escitalopram (MW = 324), as the preferential route of elimination is based on the value of the threshold MW for appreciable biliaire excretion, with man and dog being a poor and good biliairy excreter, respectively. Further, in case of hepatic clearance, substances with a low extraction ratio—also including escitalopram [5]–are prone to important interspecies variability due to large variability in the maximal metabolic capacity of P450 cytochromes[6]. The aim of the present study was to define the optimal dosing regimen of escitalopram in Beagles, by determing the elimination half-life of the product and using positron emission tomography and the radiotracer [^11^C]DASB to define the relationship between the dose and the SERT-occupancy in the brain.

## Materials and methods

### Experimental animals and study design

The study was approved by the Ethical Committee of Ghent University (EC approval 2014/125 and 2015/135). From the seven healthy adult laboratory Beagles included in the study, five of them (four male, 1 female, age 5 ± 2 years, weight 12 ± 4 kg) successfully completed the entire protocol. The other two were excluded from the study. One due to substantial head movements during the PET-acquisition and the other due to hypoproteinemia.

At first, to determine the optimal inter-dosing interval and the treatment duration to achieve steady state conditions, the plasma elimination half-life of escitalopram was examined. Because earlier experiments with racemic citalopram in Beagles strongly suggested saturable kinetics [7], one of the dogs was pretreated orally with escitalopram (0.83 mg/kg, 3 administrations/day) during four days. Thereafter, on the evening of the fourth day, a 22G catheter was placed in a cephalic vein and the last gift was replaced by an intravenous injection of 0.83 mg/kg escitalopram. Immediately after injection, venous blood samples (2–3 mL) were taken manually into heparinized syringes at several time points with increasing intervals (10, 30, 60, 90 and 120 minutes, and thereafter every two hours up to twelve hours after escitalopram injection) and collected in K_3_EDTA tubes. After centrifugation of the blood samples (5 min, 3500 rpm), the plasma fraction was separated from the blood cells and the samples were stored at -20°C until the escitalopram concentration in plasma was analyzed via LC-MS/MS at the University Medical Center in Utrecht, The Netherlands. Eventually, the elimination half-life was obtained by fitting the data with a appropriate kinetic model using GraphPad Prism 3.0 Software (GraphPad Software, inc, La Jolla, California, USA).

For the second part of the study, determining the relationship between the dose of escitalopram and the SERT-occupancy, three scans were carried-out on each dog: a structural MRI to provide anatomical information, and two [^11^C]DASB PET scans. After performing the first (baseline) PET scan, each dog was given a specific dose of escitalopram (0.50–2.5 mg/kg/day spread over several administrations/day based on the elimination half-life, estimated in part one of the study) until steady state conditions were achieved. Finally, the second PET scan was acquired five hours after the latest escitalopram administration at steady state.

On the scandays, the dogs were sedated with an i.m. injection of dexmedetomidine (375 μg/m^2^ body surface area, Dexdomitor^®^, Orion Corporation, Espoo, Finland) and transported to the PET-center of the Ghent University hospital. Once arrived, a 22G i.v. catheter was placed in a cephalic vein in order to induce general anesthesia with propofol (2–3 mg/kg, given to effect, Propovet^®^, Abbott Laboratories, Queenborough, UK). After intubation, the dogs were placed on the bed of the PET/CT scanner (sternal recumbency with the front limbs extended caudally). Anesthesia was maintained with a mixture of 1.2–1.4% isolurane (Isoflo^®^, Abbott Laboratories) in oxygen using a rebreathing system and continuous monitoring of cardiorespiratory functions by pulse oximetry and capnography was performed. The dogs were monitored during and after anaesthesia by an anesthesiologist, until fully awake.

### Radiosynthesis

The serotonin transporter ligand [^11^C]DASB was synthesized by N-methylation of the precursor N-desmethyl-DASB (50 μg, ABX, Radeberg, Germany) with [^11^C]methyl triflate using established methods [8]. This gave rise to activities of 1699 ± 752 MBq and high radiochemical purities of more than 99%. Specific radioactivities, measured with analytical HPLC, were 67 ± 28 GBq/μmol at the end of synthesis and 41 ± 11 GBq/μmol at the time of tracer injection. As all Beagles were injected with a dose of 384 ± 88 MBq, the SERT occupancy of the radiotracer, calculated via the method of Hume and colleagues (1998) and using the mean ED_50_ value of 56 nmol/kg, was 1.6 ± 0.8% [9,10].

### Imaging protocols

The MRI, performed to provide anatomical information, consisted of 3D high resolution T1-weighted images (3D MPRAGE sequence, 176 sagital slices, TR = 2250 ms, TE = 4.18 ms, TI = 900 ms, parallel acquisition method = GRAPPA with acceleration factor = 2, matrix size = 256 x 256, FOV = 220 mm, flip angle = 8°, voxel size = 1 x 1 x 1 mm^3^), and were acquired on a 3T Magnetom Trio Tim System MRI scanner (Siemens Medical Systems, Erlangen, Germany) using a phased-array spine coil and a phased-array body matrix coil.

All PET scans were acquired on a Biograph mCT 40 imaging system (Siemens, Knoxville, Tennessee, USA), consisting of a flow system with a 78 cm wide bore, LSO crystals and a True V option extending the field of view to 21.6 cm. After conducting a low dose CT survey (120 kV, 35 mAs, pitch of 0.7, 20 slices of 3 mm) for attenuation correction, 90 minutes dynamic emission recordings in list mode were initiated on bolus injection of 391 ± 69 MBq [^11^C]DASB. Emission data were corrected for dead time, scatter and random events, and subsequently reconstructed in 6 images of 10 s, 8 images of 30 s, 5 images of 120 s, and 15 images of 300s, each consisting of a 512 x 512 matrix with a voxel size of 0.797 x 0.797 x 2 mm. By using TOF and reconstructing the HD-PET data with the TrueX algorithm, the contrast was improved resulting in a 2 mm (FWHM) spatial resolution across the entire FOV.

### PET data analysis

All PET-data were analysed using the PMOD software version 3.405 (PMOD Technologies Ltd., Zurich, Switzerland). At first, to provide anatomical information, every PET image was coregistered with the corresponding MRI. Based on two dog brain atlases [11,12], six regions of interest (ROIs) were manually delineated: basal ganglia, brainstem region containing the raphe nuclei, cerebellar cortex (vermis excluded), colliculi, hippocampus, and thalamus. For each ROI, a time-activity curve (TAC) was calculated by determining the radioactivity concentration for each frame, correcting it for decay, and plotting it versus time. Based on the results of previous research [13] where multiple kinetic models were compared for the *in vivo* evaluation of [^11^C]DASB in Beagles, a non-displaceable binding potential (BP_ND_) was then calculated for each ROI via the Logan reference tissue model, thereby using the cerebellar cortex (vermis excluded) as a reference region. For every escitalopram dosing regimen allocated to one of the Beagles, the SERT-occupancy was defined as the percentage reduction of the BP_ND_ after escitalopram treatment, as compared to BP_ND_ at baseline [14]:

$$\Delta O(\%)=100\frac{(BP_{ND})baseline-(BP_{ND})posttreatment}{(BP_{ND})baseline}$$ (1)

The relationship between the dose and the occupancy of the SERT-sites was investigated by fitting the experimental data points with a hyperbolic function using Graphpad Prism 3.0. As investigated by Meyer and his colleagues [15], the minimum therapeutic dose is the one that produces 80% occupancy of the SERT-sites in the striatum.

## Results

**Fig 1:**
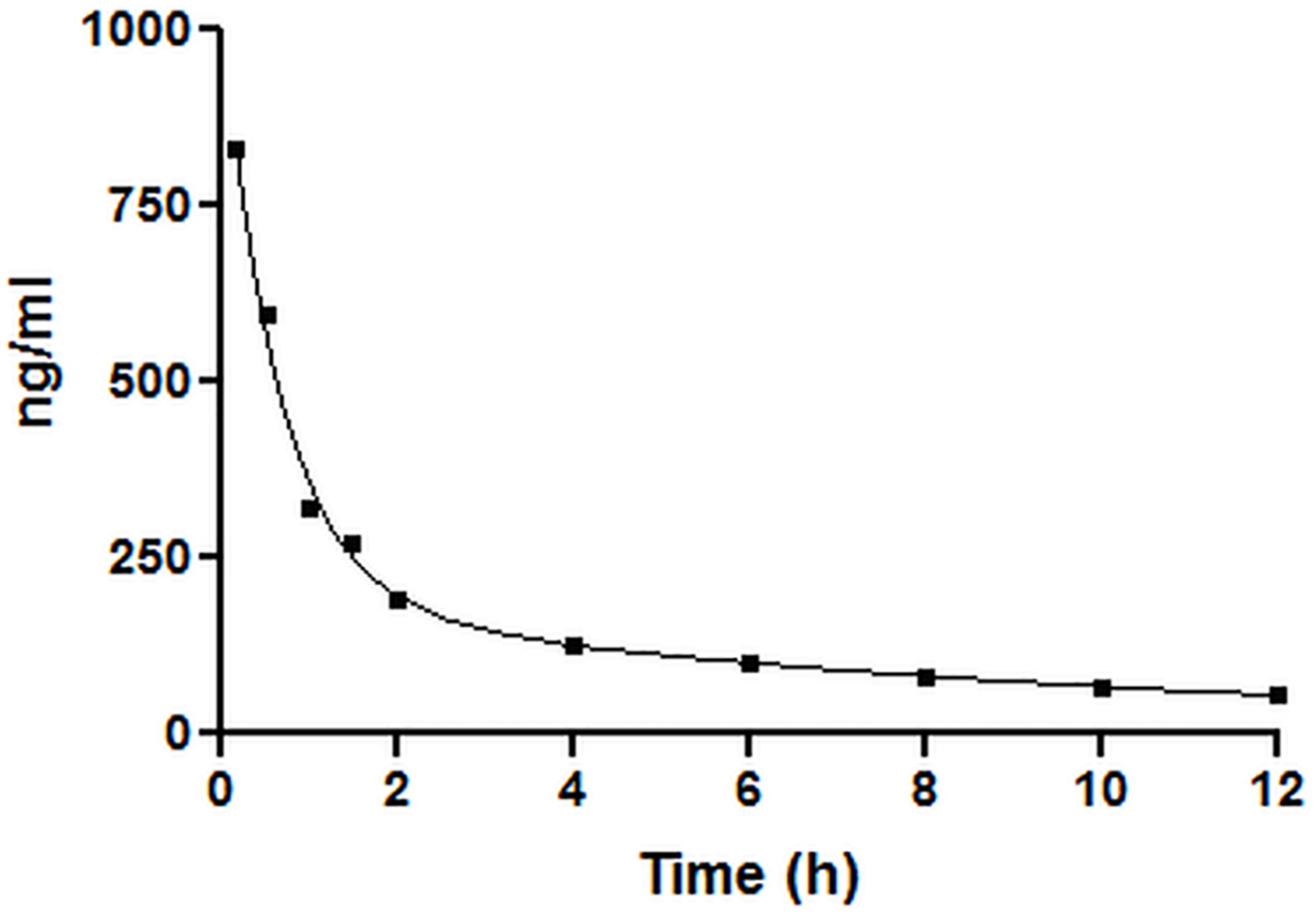
Escitalopram plasma concentration over time after IV injection of 0.83 mg/kg escitalopram. This IV injection was given 6 hours after the last gift of the preliminary oral treatment (0.83 mg/kg, 3 administrations/day, 4 days).

Preceded by an oral treatment (0.83 mg/kg, 3 administrations/day, 4 days) with escitalopram, the time course after the additional intravenous bolus (0.83 mg/kg), given six hours after the last oral dose, fitted well (R^2^ = 0.9960) with a two phase exponential decay function (Fig 1):

$$C=836.4*e^{-1.493t}+184.1*e^{-0.1034t}$$ (2)

where C represents the plasma concentration at any time t (h) after the injection.

Based on Eq (2), the terminal elimination half-life could be calculated [16]:

$$half-life=\frac{\text{ln}(2)}{0.1034h^{-1}}=6.7hours$$ (3)

Considering this half-life, for the next part of the study where the relationship between the dose and the SERT-occupancy of escitalpram was investigated, it was opted for an oral dosing frequency of three administrations a day, giving them at 7 am, 1 pm and 7 pm. Notwithstanding that the time required to reach steady state conditions equalled only 34 hours (5 x T_1/2_), a treatment period of four days was put forward before the second PET scan took place at through concentrations.

**Fig 2:**
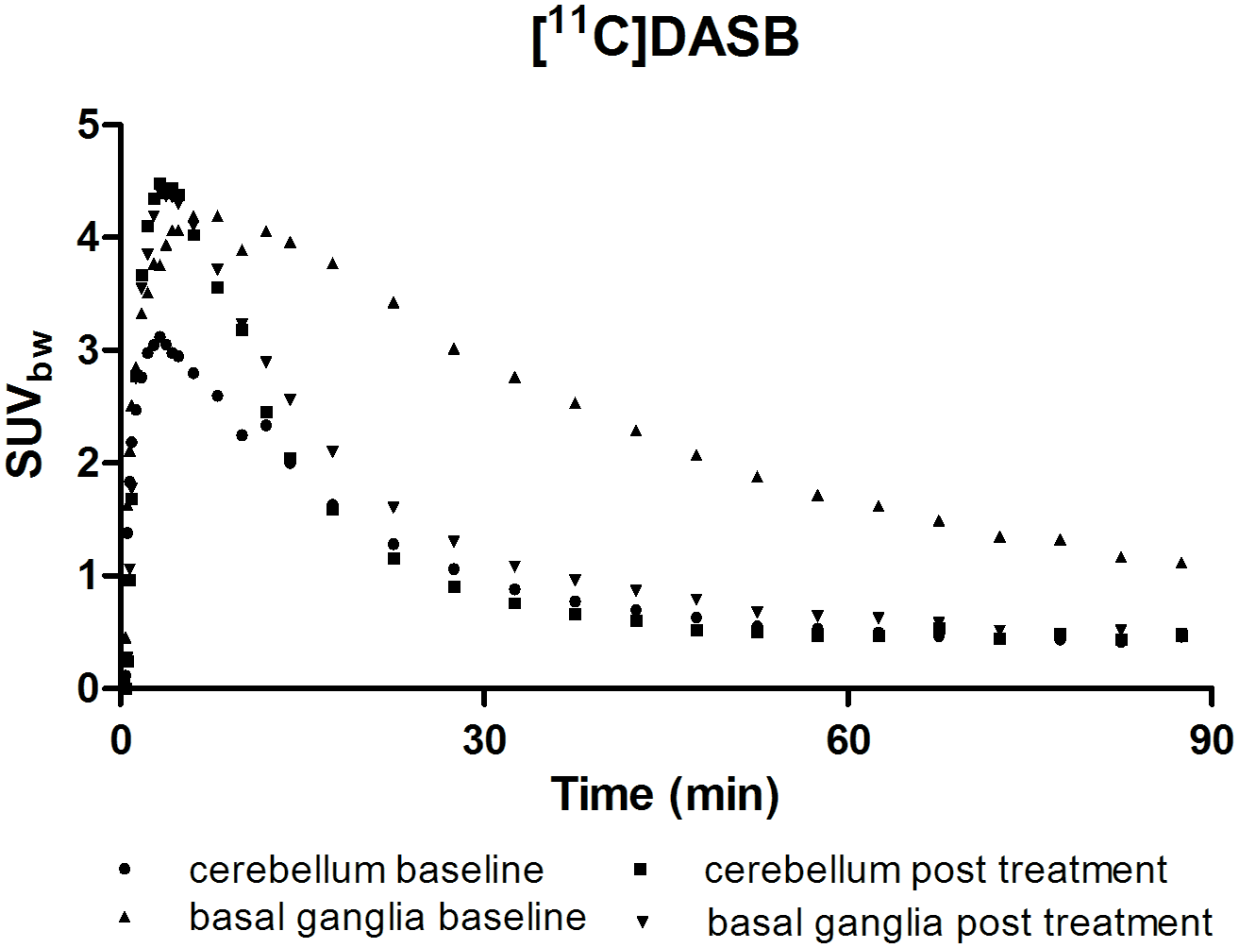
Time-activity curves in basal ganglia and reference region after IV injection of [^11^C]DASB at baseline levels and after a four day oral treatment period with escitalopram (2 mg/kg/day divided over 3 administrations).

**Fig 3:**
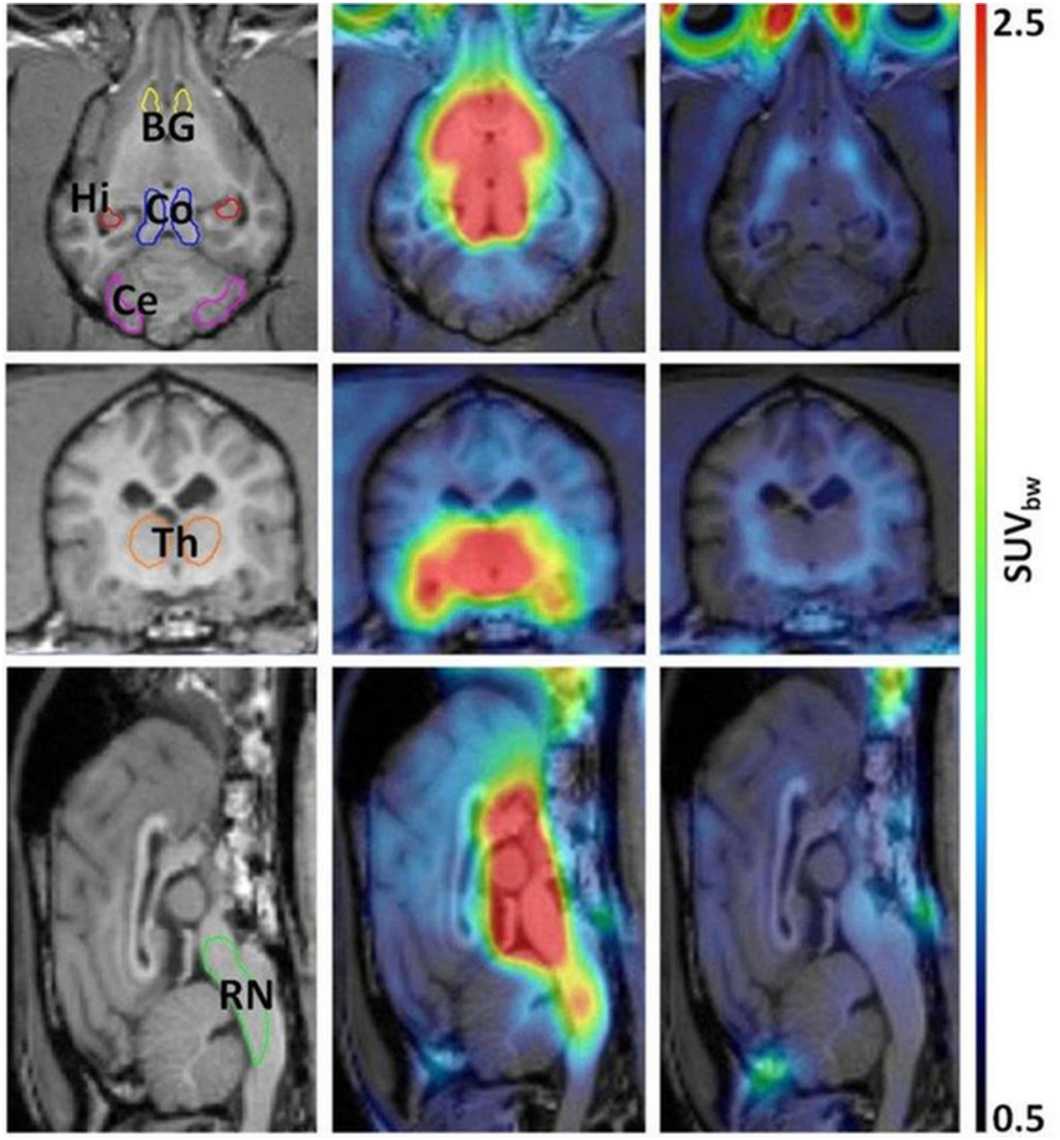
Coregistration of MRI and PET, at baseline levels and after a four day oral treatment period with 2 mg/kg/day escitalopram. The SUV_bw_ parameter is presented for each voxel on a summed PET-image between 40 and 60 minutes after IV injection of [^11^C]DASB. Regions of interest delineated on MRI: BG: basal ganglia, Ce: cerebellar cortex (vermis excluded), Co: colliculi, Hi: hippocampus, Th: thalamus, RN: brainstem region containing the raphe nuclei.

Each dog received a specific dose of escitalopram during the treatment period, ranging from 0.50 to 2.5 mg/kg/day, divided over three administrations. Figs 2 and 3 respectively represent the time-courses for the activity in basal ganglia and reference region after IV injection of [^11^C]DASB, and the SUV_bw_ parameter for each voxel on a summed PET image between 40 and 60 minutes after tracer injection, both at baseline levels and after a four day treatment period of 2 mg/kg/day.

At baseline levels, the regional distribution was consistent with the characteristic distribution of SERT in Beagles, with high SERT-availabilities in the colliculi, the thalamus and the brainstem region containing the raphe nuclei, intermediate availabilities in the basal ganglia and hippocampus, and negligible availabilities in the cerebellar cortex [17]. After escitalopram treatment, a strong decline was observed in all ROIs in comparison to the cerebellar cortex, whose shape was unchanged confirming the lack of specific binding in the latter. An overview of the BP_ND_-values, calculated via the Logan reference tissue model, before and after escitalopram treatment is presented in Table 1.

**Table 1:** BP_ND_-values, calculated with the Logan reference tissue model, for each ROI before and after escitalopram treatment.

		Basal ganglia	Hippocampus	Colliculi	Thalamus	Raphe nuclei region
**Beagle 1**	Baseline	1.932	1.421	3.646	2.933	1.875
	0.5 mg/kg/day	1.630	1.026	2.876	2.066	1.565
**Beagle 2**	Baseline	2.058	1.465	4.732	2.409	2.068
	0.75 mg/kg/day	0.771	0.545	2.077	1.028	0.889
**Beagle 3**	Baseline	1.827	1.455	3.723	2.651	2.664
	1.3 mg/kg/day	0.592	0.508	1.503	0.883	0.934
**Beagle 4**	Baseline	1.511	0.910	3.881	2.100	2.016
	2.0 mg/kg/day	0.197	0.062	0.339	0.266	0.341
**Beagle 5**	Baseline	1.973	1.515	3.119	2.368	1.374
	2.5 mg/kg/day	0.317	0.184	0.378	0.378	0.218

**Fig 4:**
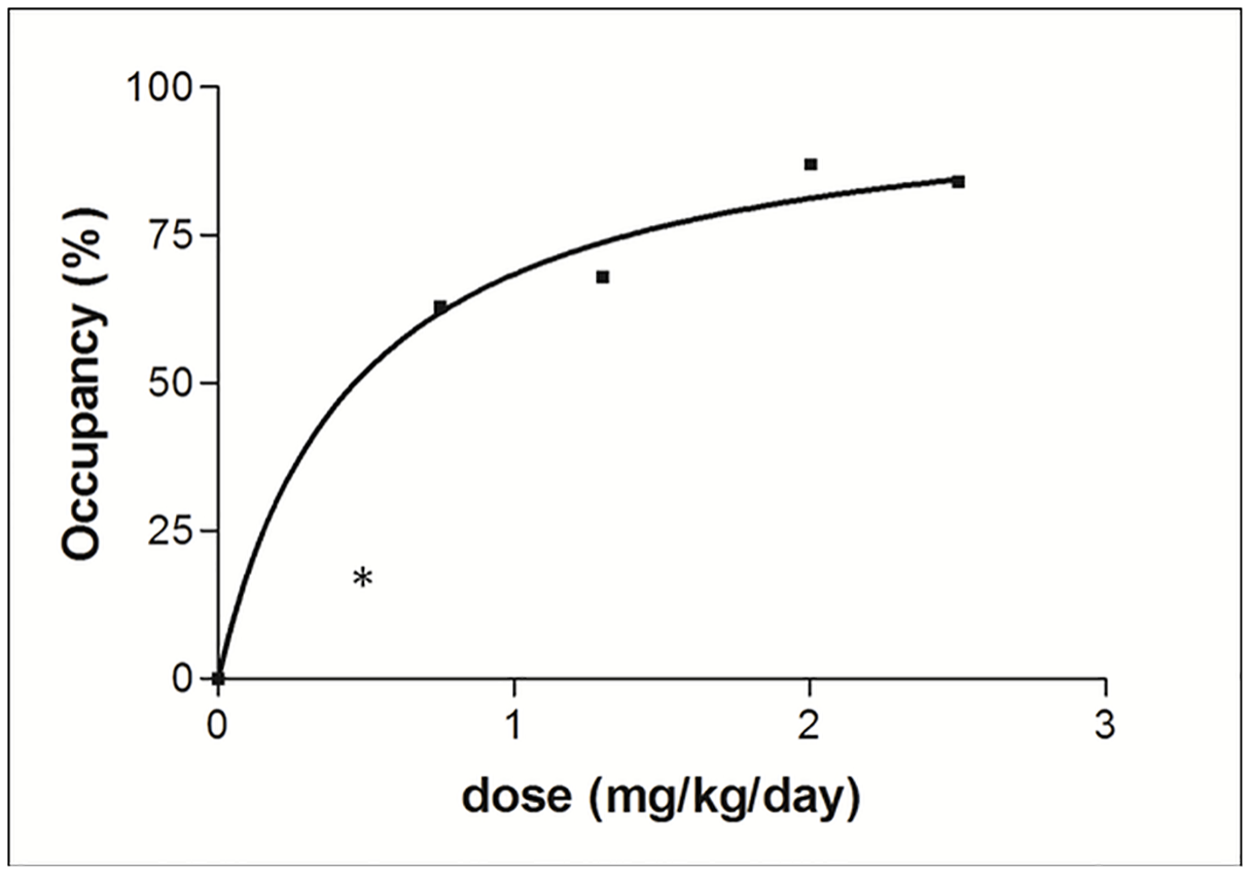
Relationship between the dose of escitalopram and the SERT-occupancy measured in the basal ganglia. R^2^ represents the nonlinear regression fit of the hyperbola; K_D_^app^ represents the required dose to occupy 50% of the SERT sites. * represents the excluded experimental data point for the fitting.

The relationship between the dose of escitalopram and the striatal SERT occupancy was examined by fitting the experimental data points with a one side binding hyperbola (Fig 4). Fitting all data points resulted however in a rather unsatisfying non linear regression fit (R^2^) of 0.8498, which was due to the very low SERT-occupancy of 16% after treatment with 0.5 mg/kg escitalopram a day. As our main interest was accurately defining the required dose to occupy 80% of the SERT-binding sites, it was decided to exclude this lowest experimental data point, which resulted in a much better fit (R^2^ = 0.9863), especially at higher occupancy values required for therapeutic efficacy.

This hyperbolic equation could be mathematically described as:

$$Occupancy(\%)=\frac{Occ_{\text{max}}*D}{K_{D}^{app}+D}$$ (4)

where Occ_max_ represents the maximal occupancy (100%), D represents the dose of escitalopram (mg/kg/day) during the treatment period, and K_D_^app^ represents the required dose at which 50% of the SERT-sites are occupied [18]. As the K_D_^app^ value was calculated to be 0.4630 mg/kg/day, the minimal dose to occupy 80% of the SERT-sites in the basal ganglia and elicit therapeutic effects equalled 1.85 mg/kg/day. Besides 80% striatal occupancy, this dosing regimen also resulted in a similar occupancy in the other ROIs included in this study, more specific 81% in the hippocampus, 78% in both the colliculi and the thalamus, and 77% in the brainstem region containing the raphe nuclei.

## Discussion

Dose occupancy studies aim at optimising the dosing regimen of a particular substance in a particular species, thereby avoiding doses that are too low, and therefore ineffective, or doses that are too high, potentially causing side effects. In this respect, attention should be given to the concentration of the didesmethylmetabolite of escitalopram (S-DDCT), which in dogs is the main plasma metabolite. Although no original data were published with regard to this issue, it was observed that, in dogs, the QT interval on an electrocardiogram can be affected by concentrations of DDCT above 300 ng/ml [19]. Based on the performed GC-MSMS analysis of S-DDCT in plasma after therapy with 1.3 and 2.0 mg/kg/day (data not presented), the S-DDCT resulting from the suggested dosing regimen of 1.85 mg/kg/day, divided over three administrations, is estimated to equal 290 ng/ml. Therefore in case of long-term therapy, regularly cardiac screening is recommended.

During the second part of the study it was observed that treatment with 0.5 mg/kg escitalopram per day resulted in an occupancy of only 16%. A likely explanation could be that treatment with a low dose of 0.5 mg/kg/day does not result yet in saturated kinetics, entailing a shorter elimination half-life of escitalopram and thus a lower occupancy of the SERT binding sites. Because a similar phenomenon has previously also been reported after administration of different doses of citalopram in dogs [7], it was decided to exclude this value during the fitting of the hyperbola. This implies however that steady state occupancy values after treatment with low escitalopram doses (≤ 0.5 mg/kg/day) could not accurately be predicted based on the KD,app calculated in this study. However, since it is reported that minimal 80% occupancy of the serotonin transporters is required to provoke a pharmacological effect, the impact of this limitation is questionable.

As recommended by Meyer [20], it was opted to use the basal ganglia as the principal region of interest for the occupancy measurements. This because of its large structure, its reported homogenous uptake of [^11^C]DASB, and its reported excellent consistency of test-retest measurements in humans. Although several clinical studies report nonhomogenous SERT-occupancies in the brain, more specific higher occupancies in the midbrain [20,21], and lower occupancies in the thalamus [20], these findings were not reproduced in the present study. The occupancy under steady state conditions of the suggested dosing regimen of all included ROIs where within 4% of the occupancy in the basal ganglia.

Due to its short elimination half-life in Beagles—6.7 hours compared to 27–33 hours in humans [22]–a dosing frequency of three administrations a day is required. Therefore, when it comes to therapy compliance, escitalopram treatment is not favored over treatment with fluoxetine, which is currently the only registered SSRI for veterinary use and benefits from its once daily administration. Despite this, under certain circumstances, therapy with escitalopram might still be a better choice. At first, although SSRIs are similarly efficacious for the treatment of depression, it is not predictable which SSRI will give the best results for a given patient [23]. Therefore, in case of no or insufficient response of a dog to fluoxetine, escitalopram might be a promising alternative, especially because of its additional interaction with allosteric binding sites at the SERT. Further, although all SSRIs are extensively biotransformed by the P450 system, it has been reported that fluoxetine, and also fluvoxamine and paroxetine, also significantly inhibit one or more of the P450 enzymes [23], which may potentially result in a substantial disturbance of the metabolism of other drugs in case of concurrent drug intake. As this P450 inhibition has not been observed with escitalopram, such therapy might be recommended in case the dog is already under medical treatment for other disorders or diseases. Finally, as escitalopram contains the highest selectivity among all SSRIs, >1000 compared to its nearest target [24], it can be put forward as the most suitable SSRI to use in research on SERT.

## Conclusion

This study aimed to estimate the optimal dosing regimen of escitalopram in Beagles. Hereby the elimination half-life was determined to be 6.7 hours, suggesting a dosing schedule of three administrations a day, and based on a miminum 80% striatal occupancy for therapeutic effect, a dose of 1.85 mg/kg/day is required. Under certain circumstances the use of escitalopram can be preferred over the use of the already for veterinary use registered fluoxetine, however, in case of long-term treatment, regularly cardiac screening is recommended.

## Supporting information

S1 File Supplemental data concerning [^11^C]DASB administration, clearance of escitalopram from plasma, and regional dose occupancy values. (PDF)
