# Supplementary material for: Estimation of the optimal dosing regimen of escitalopram in dogs: A dose occupancy study with [11C]DASB
Source: PLoS One. 2017 Jun 23;12(6):e0179927. doi: 10.1371/journal.pone.0179927 (PMC5482480; doi:10.1371/journal.pone.0179927)
Supplement: S1 File — (PDF) [file pone.0179927.s001.pdf]

Supplemental data concerning [^{11}C]DASB administration, clearance of escitalopram from plasma, and regional dose occupancy values

[^{11}C]DASB administration

			Baseline		Post treatment	
**Beagle**	**Weight (kg)**	**Escitalopram dose (mg/kg/day)**	**[^{11}C]DASB activity injected (MBq/kg)**	**% SERT occupancy by [^{11}C]DASB**	**[^{11}C]DASB activity injected (MBq/kg)**	**% SERT occupancy by [^{11}C]DASB**
1	7	0.5	0.98	1.1	1.21	3.3
2	12	0.75	0.88	1.2	0.80	1.5
3	14	1.3	1.00	1.5	0.74	0.6
4	18	2	0.79	1.3	0.70	1.1
5	7	2.5	1.09	2.1	1.39	2.2

Plasma activity curve from which the elimination half-life was determined

(Fig 1 in manuscript: “escitalopram plasma concentration over time after IV injection of 0.83 mg/kg escitalopram. This IV injection was given 6 hours after the last gift of the preliminary oral treatment 0.83 mg/kg, 3 administrations/day, 4 days”)

Hours after latest (i.v.) escitalopram injection	Plasma escitalopram concentration (ng/ml)
0.17	826
0.50	595
1.0	319
1.5	269
2	190
4	122
6	100
8	81
10	65
12	53

Dose occupancy study

	% occupancy of SERT by escitalopram				
**Dose (mg/kg/day)**	**Basal ganglia**	**Hippocampus**	**Colliculi**	**Thalamus**	**Brainstem**
0.5	16	28	21	30	17
0.75	63	63	56	57	57
1.3	68	65	60	67	65
2.0	87	93	91	87	83
2.5	84	88	88	84	84
